# Osteoglycin inhibition by microRNA miR-155 impairs myogenesis

**DOI:** 10.1371/journal.pone.0188464

**Published:** 2017-11-21

**Authors:** Paula Paccielli Freire, Sarah Santiloni Cury, Grasieli de Oliveira, Geysson Javier Fernandez, Leonardo Nazario Moraes, Bruno Oliveira da Silva Duran, Juarez Henrique Ferreira, César Seigi Fuziwara, Edna Teruko Kimura, Maeli Dal-Pai-Silva, Robson Francisco Carvalho

**Affiliations:** 1 Department of Morphology, Institute of Biosciences, São Paulo State University, Botucatu, São Paulo, Brazil; 2 Department of Cell and Developmental Biology, Institute of Biomedical Sciences, University of Sao Paulo, Sao Paulo, Brazil; University of Birmingham, UNITED KINGDOM

## Abstract

Skeletal myogenesis is a regulated process in which mononucleated cells, the myoblasts, undergo proliferation and differentiation. Upon differentiation, the cells align with each other, and subsequently fuse to form terminally differentiated multinucleated myotubes. Previous reports have identified the protein osteoglycin (Ogn) as an important component of the skeletal muscle secretome, which is expressed differentially during muscle development. However, the posttranscriptional regulation of Ogn by microRNAs during myogenesis is unknown. Bioinformatic analysis showed that miR-155 potentially targeted the Ogn transcript at the 3´-untranslated region (3´ UTR). In this study, we tested the hypothesis that miR-155 inhibits the expression of the Ogn to regulate skeletal myogenesis. C2C12 myoblast cells were cultured and miR-155 overexpression or Ogn knockdown was induced by transfection with miR-155 mimic, siRNA-Ogn, and negative controls with lipofectamine for 15 hours. Near confluence (80–90%), myoblasts were induced to differentiate myotubes in a differentiation medium. Luciferase assay was used to confirm the interaction between miR-155 and Ogn 3’UTR. RT-qPCR and Western blot analyses were used to confirm that the differential expression of miR-155 correlates with the differential expression of myogenic molecular markers (Myh2, MyoD, and MyoG) and inhibits Ogn protein and gene expression in myoblasts and myotubes. Myoblast migration and proliferation were assessed using Wound Healing and MTT assays. Our results show that miR-155 interacts with the 3’UTR Ogn region and decrease the levels of Ogn in myotubes. The overexpression of miR-155 increased MyoG expression, decreased myoblasts wound closure rate, and decreased Myh2 expression in myotubes. Moreover, Ogn knockdown reduced the expression levels of MyoD, MyoG, and Myh2 in myotubes. These results reveal a novel pathway in which miR-155 inhibits Ogn expression to regulate proliferation and differentiation of C2C12 myoblast cells.

## Introduction

Skeletal myogenesis is a multistep process in which myoblasts are withdrawn from the normal cell cycle to subsequently fuse and terminally differentiate into multinucleated myotubes [1]. This process is highly regulated by the myogenic transcription factors MyoD, Myf5, myogenin, MRF4, and Mef2 that coordinate the expression of muscle-specific genes (reviewed in [2,3]). Several studies have demonstrated that skeletal myogenesis is also regulated by microRNAs (miRNAs) [4], a class of short non-coding RNAs that post-transcriptionally regulate gene expression by translational repression or the degradation of protein-coding mRNAs (reviewed in [5,6]).

Muscle-specific miRNAs (miR-1, -133, -206, -208, -208b, and -499) have been demonstrated to participate in relevant physiological and pathological skeletal muscle processes, such as myogenesis, regeneration, hypertrophy, and muscular dystrophy [7–13]. Moreover, miR-208b and miR-499 are a part of a myomiR network that controls Myh2C expression, fiber-type and muscle performance [12]. Emerging evidence also supports the involvement of non-muscle miRNAs in muscle hypertrophy [14], atrophy [15–17], and myogenesis [18,19]. Among these non-muscle-specific miRNAs, miR-155 has a distinctive expression pattern associated with different types of primary muscle disorders [7] and facilitates skeletal muscle regeneration by balancing pro- and anti-inflammatory macrophages [20,21]. It has also been demonstrated that miR-155 controls myoblast differentiation by post-transcriptionally inhibition of the Mef2a expression [22]. However, the fact that a single miRNA has the capacity to repress multiple target transcripts [23,24], in a coordinated manner, raises the need to unveil novel and potential miR-155 target transcripts and their functions during skeletal myogenesis. Interestingly, a previously integrative miRNA-mRNA analysis predicted several target mRNAs in skeletal myocyte differentiation and identified osteoglycin (Ogn) among the potential miR-155 target transcripts [25]. These results further reinforce the hypothesis that miR-155 may repress additional target transcripts, including Ogn, during myogenesis.

Ogn is an extracellular matrix component belonging to a small leucine-rich proteoglycan gene family [26–28]. This protein is abundant in connective tissues [27,29] and it was first described as involved in bone formation [30,31]. In the left ventricle, this protein is an important regulator of cardiomyocyte hypertrophy with a prominent role in collagen fibrillogenesis [32,33]. Ogn is also produced by skeletal muscle cells and induces bone anabolic effects [31]. Most importantly, Ogn is considered a crucial component of the secretome for skeletal muscle cells because Ogn siRNA mediated knockdown decreases myoblast differentiation as indicated by the Muscle Creatine Kinase MCK-Luc reporter gene system [34]. Moreover, data from GEO Profiles (Profile GDS233 / 99549_at) shows an increase in Ogn expression during muscle regeneration (S1 Fig). However, the post-transcriptional regulatory mechanisms of Ogn that may affect migration, proliferation, and differentiation during myogenesis need to be clarified further. Thus, in this study, we tested the hypothesis that miR-155 inhibits Ogn expression to regulate skeletal myogenesis.

## Material and methods

### Cell culture and muscle differentiation

C2C12 mouse myoblasts (ATCC® CRL-1772^TM^) were cultured in a growth medium (GM) consisting of Dulbecco's modified Eagle's medium (DMEM, Thermo Fisher Scientific, USA) supplemented with 1% Penicillin–Streptomycin (Thermo Fisher Scientific, USA) and 10% fetal bovine serum (Thermo Fisher Scientific, USA) at 37°C and 5% CO2. After transfection, near-confluent cells (80% to 90%) were induced to differentiate in a differentiation medium (DM), consisting of DMEM plus 2% horse serum (Thermo Fisher Scientific, USA) and 1% Penicillin–Streptomycin solution for 5 days. All the experiments were carried out using at least three independent replicates per group.

### Oligonucleotides and transfection

The miR-155 mimic (Thermo Fisher, mirVanaTM miRNA Mimic, code: 4464066; MC13058—MC10203), the small interfering RNA siRNA against Ogn (Thermo Fisher, Silencer® Select siRNA s70945 and s70947), the respective negative controls (Thermo Fisher, mirVanaTM miRNA Mimic Negative Control, code: 4464058 and Silencer® Select Negative Control No. 1 siRNA) formed a complex with Opti-MEM reduced serum medium (Thermo Fisher Scientific, USA) before transfection. C2C12 myoblasts transfections were performed with RNAiMAX lipofectamine (Thermo Fisher Scientific, USA) combined with 30 nM of each oligonucleotide for 15 hours, when the cells were approximately 80% confluent (day 0). Differentiation was induced 24 hours post-transfection and is shown as day 1 (S2 Fig). For all experiments, the cells were analyzed from day 0 up to day 5, then described in the following topics.

### RNA isolation

The total RNA was extracted from C2C12 cells using a TRIzol reagent (Thermo Fisher Scientific, USA), as recommended by the manufacturer. RNA concentration and quality were assessed using a NanoVue Plus Spectrophotometer (GE Healthcare, USA). RNA quality was also assured by the RNA integrity number (RIN) obtained from an analysis of ribosomal RNAs based on microfluidics using the 2100 Bioanalyzer system (Agilent, USA). Only RNA samples with a A260/280 ratio of 1.8–2.0, a A260/230 ratio > 2.0, and a RIN > 9 was used for subsequent analysis.

### RT-qPCR

MicroRNA cDNA was synthesized from 1μg total RNA samples using specific miRNA stem-loop primers and TaqMan MicroRNA reverse Transcription Kit (Thermo Fisher Scientific, USA). Total RNA samples were also reverse transcribed into cDNA using the High Capacity RNA-to-cDNA Master Mix (Thermo Fisher Scientific, USA). miRNA and mRNAs qPCR analysis were performed in a 15 μl reaction (TaqMan™ Gene Expression Master Mix for miRNAs, and Power SYBR™ Green Master Mix for mRNAs; Thermo Fisher Scientific, USA), as described by the manufacturer, and run on a QuantStudio™ 12K Flex System (Thermo Fisher Scientific, USA) using the following cycle conditions: 95°C for 10 mins followed by 40 cycles of 95°C for 15 secs and 60°C for 1 min. The oligonucleotides for mRNAs are listed in S1 Table, and the miRNAs TaqMan Assays (Thermo Fisher Scientific, USA) used were: miR-155 (Assay ID #002571), and U6 (Assay ID #001973). Finally, the raw data was retrieved and imported into the Expression Suite Software v1.0.3 (Thermo Fisher Scientific, USA). Small RNA MammU6 and Rpl13a were used as reference genes to normalize the miRNA and mRNA data, respectively. These reference genes were selected based on geNorm calculations [35], and the relative expression levels of these reference genes were similar between control and miR-155 mimic treated samples as indicated by the Ct value reported after analysis of raw qPCR data (S2 Table). Relative quantification of miRNA and mRNA expression was evaluated using the 2^-ΔΔCT^ method [36]. Cutoffs for significant changes were set at fold-change >1.5 and p-value ≤ 0.05.

### DNA constructs

Using a TargetScan algorithm (http://www.targetscan.org), the segment containing a miR-155 binding site on Ogn 3’UTR was generated. The oligonucleotides sequence Ogn-Fw-wt and Ogn-Rev-wt were flanked by XhoI and XbaI restriction enzymes sites (S3 Fig). Similarly, the miR-155 binding site with a mutated seed region was generated by annealing of Ogn-Fw-Mut and Ogn-Rev-Mut sequences. To form the plasmids pmiRGlo-OGN-3’UTR-wt and–Mut, both segments were cloned downstream from the luciferase reporter gene in the pmirGLO Dual-Luciferase miRNA Target Expression Vector (Promega, USA).

### Luciferase reporter assays

Using 12-well plates with C2C12 (ATCC® CRL-1772^TM^) and NIH/3T3 (ATCC® CRL-1658™) cells at a density of 10^5^ cells/well, the plasmids pmiRGlo-OGN-3’UTR-wt or–Mut, and miR-155 mimic were transfected using a RNAiMAX lipofectamine (Thermo Fisher Scientific, USA). As a control, the co-transfections of anti-miR-155 or a negative control was used. The cells were transfected for 10 hours, washed with phosphate- buffered saline (PBS) 48 hours after transfection, lysed with Passive Lyses buffer (Promega, USA), and the luciferase activity was obtained using DualGlo Luciferase Assay System (Promega, USA) according the manufacturer’s instructions.

### Cell proliferation assay

The viability of the proliferating C2C12 myoblasts, after transfections, was determined using a 3-(4.5-dimethylthiazol-2-yl)-2.5- diphenyltetrazolium bromide (MTT) assay (Sigma, USA). The cells were seeded into 96-well plates, and at 0 hours, 12 hours, 24 hours, 36 hours, and 48 hours, 180 uL of MTT solution (0.5 mg/mL) in phosphate- buffered saline (PBS) was added to each well. After each time-point, the plates were incubated for 4 hours at 37°C. The reaction product, precipitated formazan, was solubilized in 100% dimethyl sulfoxide (100 mL/well) and the absorbance was measured using an Infinite 200 PRO Teca multidetection micro plate reader (TECAN) at a wavelength of 570 nm.

### Wound healing assay

Wound healing assay was used in several studies with the C2C12 lineage [37–41]. C2C12 cells were plated in 6-well plates and cultured in DMEM and, after transfection, were cultivated at 37°C and 5% CO2 until they reached 90–100% confluence. Subsequently, a vertical single-line scratch was mechanically generated at the highest diameter of each well-plate, in the same position for all replicates, using a 200-μl plastic tip [scratch wound length: 784 ± 107 μm (mean ± SD, n = 36)]. Cell debris was removed using two PBS washes, and 2mL of DMEM supplemented with 2% fetal bovine serum was added to each well, following the protocol previously described [42]. The wound open area was photographed and analyzed at 0 hours, 12 hours, 24 hours and 48 hours, and the data was reported as percentage wound healing with the following equation: % wound healing = [100 − (wound area at T_nh_/wound area at T_0h_)] × 100, where T_0h_ is the time point immediately after the scratch. The wound closure rate was also calculated by averaging out the difference in the normalized area between the first four time points and then normalized to the controls.

### Western blot analysis

C2C12 cells were harvested in PBS after transfections, and whole-cell lysates were prepared by re-suspending cell pellets in ice-cold RIPA buffer (Radio-Immunoprecipitation Assay, Sigma, USA) containing 1% PMSF (protease inhibitor), and incubating the suspension on ice for 30 mins. Supernatant lysates were collected following centrifugation (8.000 x g) for 10 mins at 4°C. Supernatant lysates protein concentration was determined using a Bradford protein assay kit according to the manufacturer’s instructions (Bradford3, Bio-Rad Laboratories, USA). Subsequently, Lammeli buffer (Sigma, USA) was added to each sample and boiled at 100°C for 10 mins. Proteins were subjected to SDS-PAGE in 10% polyacrylamide gels, according the protein molecular weight. After electrophoresis, proteins were electrotransferred to nitrocellulose membranes (Bio-Rad, USA). The blotted membranes were blocked with 5% non-fat dry milk in TBS buffer containing 0.5% Tween 20 (TBST) for 2 hours at room temperature, and then incubated overnight at 4–8°C with specific antibodies against Ogn (Santa Cruz Biotechnology, USA; 1:1000 dilution), MYH2 (Santa Cruz Biotechnology, USA; 1:500 dilution), α-tubulin (Santa Cruz Biotechnology, USA; 1:200 dilution), and GAPDH (ABR, Affinity BioReagents, USA; 1:2500 dilution). Primary antibody binding was detected with peroxidase conjugated secondary antibodies (rabbit or mouse, depending on the protein, for 2 hours at room temperature), developed using enhanced chemiluminescence (Amersham Biosciences, USA), and detected by autoradiography in ImageQuant™ LAS 4000 (GE Healthcare, USA). Quantification analyses of blots were performed by ImageJ software. Targeted bands were normalized to GAPDH or α-tubulin expressions.

### Immunostaining

C2C12 myotubes cultured in 6-well plates were fixed in 4% paraformaldehyde for 15 mins, washed with PBS and 0.1% TritonX-100 (Sigma, USA), and blocked with 3% BSA, 1% glycine, 8% fetal bovine serum in PBS and 0.1% TritonX-100 for 1 hour at room temperature. Subsequently, the cells were incubated with primary (Myh2 and Ogn) antibodies overnight at 4°C and, after washing, the cells were incubated with secondary antibodies for 1 hour at room temperature and counter stained with DAPI. All images were acquired at room temperature from scanning confocal microscope TCS SP5 (Leica Microsystems, UK). Myh2 pixels were counted using TCS SP5 (software Leica Microsystems, UK). Total nuclei, myotubes nuclei, and the myotubes area were measured using ImageJ software. The fusion index was determined as (total myotube nuclei/total nuclei) x 100.

### Statistical analysis

The normality test was performed using the software GraphPad Instat Demo. All experimental data was normally distributed and expressed as the mean ± standard deviation (SD). Data was analyzed using the Student’s t-test (using GraphPad Prism 6) to establish a significant value between data points. The values of P<0.05 were considered statistically significant.

## Results

### Identification of osteoglycin (Ogn) as a novel transcriptional target of miR-155

**Fig 1:**
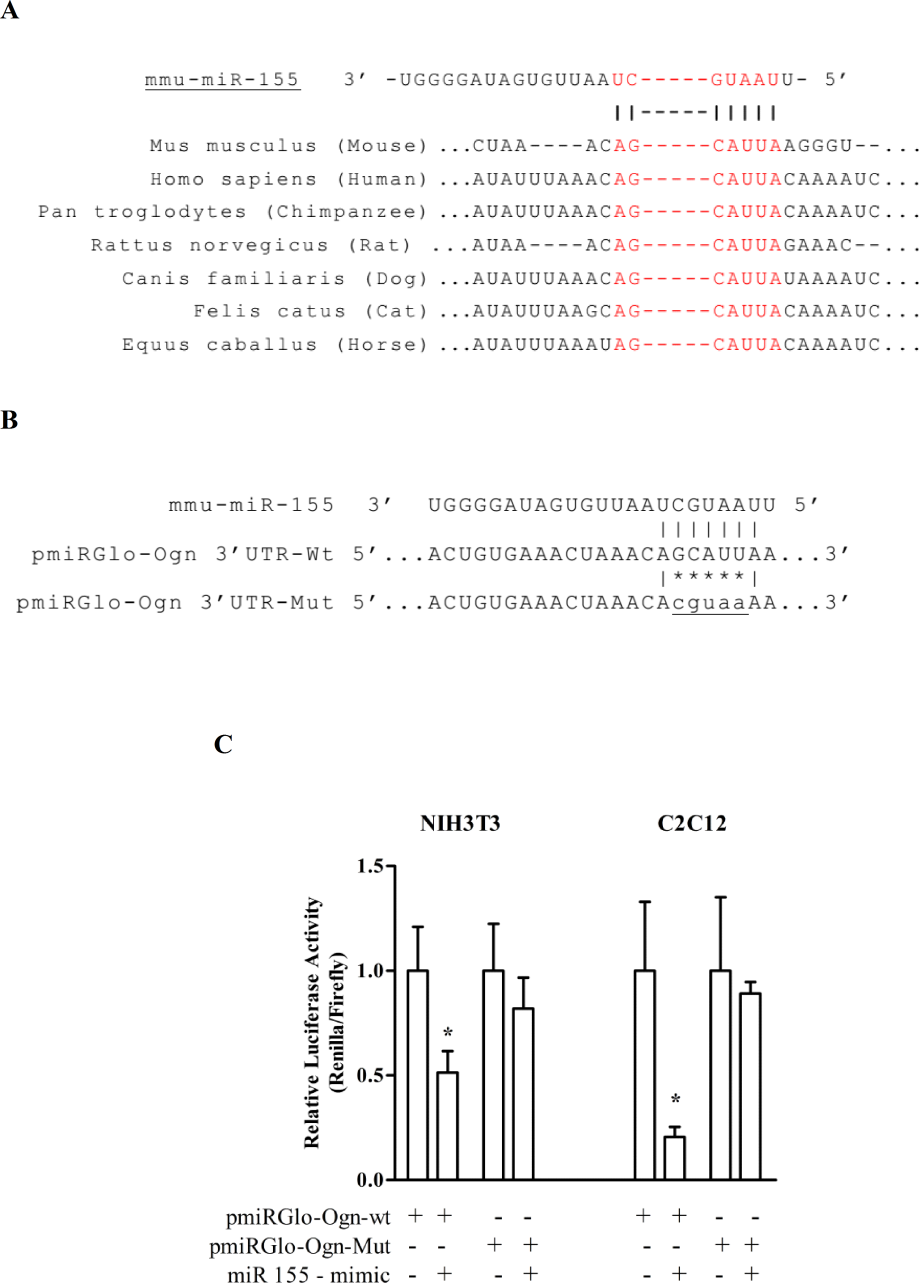
Identification of osteoglycin (Ogn) as a novel transcriptional target of miR-155. (A) Conserved seed-matched sequences in the Ogn 3´UTR are shown in red in the TargetScan. (B) The predicted binding site of miR-155 on Ogn 3’UTR was cloned in pmiRGlo plasmid, generating pmiRGlo-Ogn-3’UTR-wt plasmid. A plasmid containing the mutated binding site, shown as asterisks, was used as a control. (C) The respective luciferase reporter plasmid was transfected alone (Ogn-wt, Ogn-Mut); co-transfected with miR155-mimic (Ogn-wt + miR-155, Ogn-Mut + miR-155) in both C2C12 and NIH/3T3 cells. The data represents the average of three independent experiments, and bars represent the standard deviation. The statistical significance was analyzed using the Student’s t-tests. *P < 0.05.

We identified Ogn transcript (NM_008760.4) as a potential target for the miR-155 target site on its 3‘UTR through five different established miRNA prediction programs (miRanda [43], TargetScan [44], RNAhybrid [45], miRWalk [44], and RNA22 [46]). Among the different algorithms, the miRanda prediction presented a good miRSVR score (-0.5421), which is a regression model that calculates the weighted sum of a number of sequences and the context features of the predicted miRNA-mRNA duplex [47]. Interestingly, a minimum free energy −23.6 kcal/mol was found in a miR-155 binding site in Ogn using the software RNAhybrid, which means that it has a stable structure and may be a potential target site for miR-155 in Ogn mRNA. According to TargetScan, the matched region of the miRNA-mRNA interaction is conserved in different species, such as human, chimpanzee, rat, dog, cat, and horse (Fig 1A). Moreover, the miR-155 sequence is highly conserved in various species based on the alignment of a combined set of all previously reported mature miRNAs from miRBase database (S4 Fig). Together, this data suggests that Ogn is a putative regulatory target of miR-155.

To test whether miR-155 can regulate the Ogn expression through the binding to its 3’UTR, a wild type and a mutated binding site of miR-155 on Ogn 3’UTR were cloned into a luciferase reporter plasmid, generating the plasmids pmiRGlo-Ogn-3’UTR-wt and -Mut, respectively (Fig 1B). The co-transfection of pmiRGlo-Ogn-wt with miR 155-mimic led to a reduction in the luciferase activity in both the C2C12 and NIH/3T3 cell lines (Fig 1C). In contrast, miR-155 was not able to bind on to the mutated construct in these same cells.

### miR-155 represses Ogn and alters the expression levels of myogenesis molecular markers

**Fig 2:**
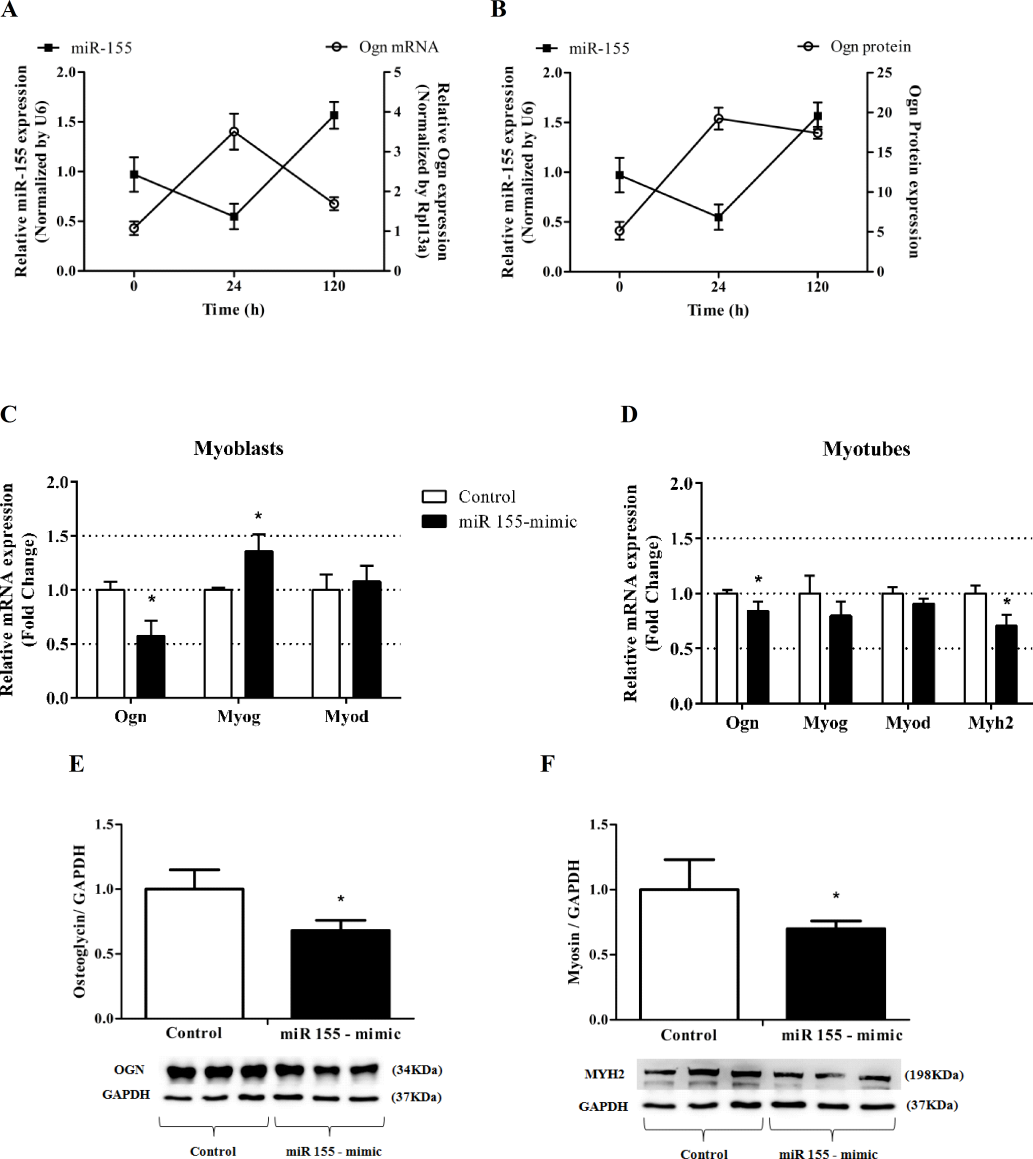
miR-155 represses Ogn and alters the expression levels of myogenesis molecular markers. (A) mRNA levels of Ogn and miR-155 in C2C12 myoblasts (0 hours) and myotubes (24 hours and 120 hours, post differentiation). (B) Protein levels of Ogn and mRNA levels of miR-155 in C2C12 myoblasts (0 hours) and myotubes (24 hours and 120 hours, post differentiation). (C) mRNA levels of Ogn, Myog, and Myod in myoblasts after miR155-mimic transfection. (D) mRNA levels of Ogn, Myog, Myod, and Myh2 in myotubes after miR155-mimic transfection. Protein levels of Ogn (E) and Myh2 (F) normalized by Gapdh in myotubes transfected with miR155-mimic. mRNA and miRNA RT-qPCR data is presented as fold change (2^-ΔΔCt^) relative to Rpl13a or U6, respectively. Data represents the average of three independent experiments, and bars represent the standard deviation. The statistical significance was analyzed using the Student’s t-tests. *P < 0.05. Ogn: osteoglycin; Rpl13a: ribosomal protein L13A; Myog: myogenin; Myod: myogenic differentiation 1; Myh2: Myosin heavy chain II; Gapdh: glyceraldehydes-3-phosphate dehydrogenase.

Firstly, we detected that miR-155 expression levels are inversely associated with Ogn transcript and protein levels in C2C12 myoblasts (0 hours) and myotubes (24 hours and 120 hours, post differentiation) (Fig 2A and 2B). Next, we asked whether miR-155 mediates the repression of Ogn expression with a functional consequence in skeletal muscle cells. To test this, we transfected a synthetic miR-155 mimic into C2C12 myoblasts. The miR-155 overexpression repressed Ogn transcript levels and increased Myog mRNA expression levels with no effect on Myod mRNA in myoblasts (Fig 2C). Moreover, miR-155 overexpression decreased Ogn transcript and protein levels in myotubes (Fig 2D and 2E). The transcript and protein levels of the myogenic differentiation marker Myh2 decreased in differentiating myoblasts suggesting that miR-155 represses myoblast differentiation (Fig 2D and 2F).

### Ogn alters the expression levels of myogenesis molecular markers

**Fig 3:**
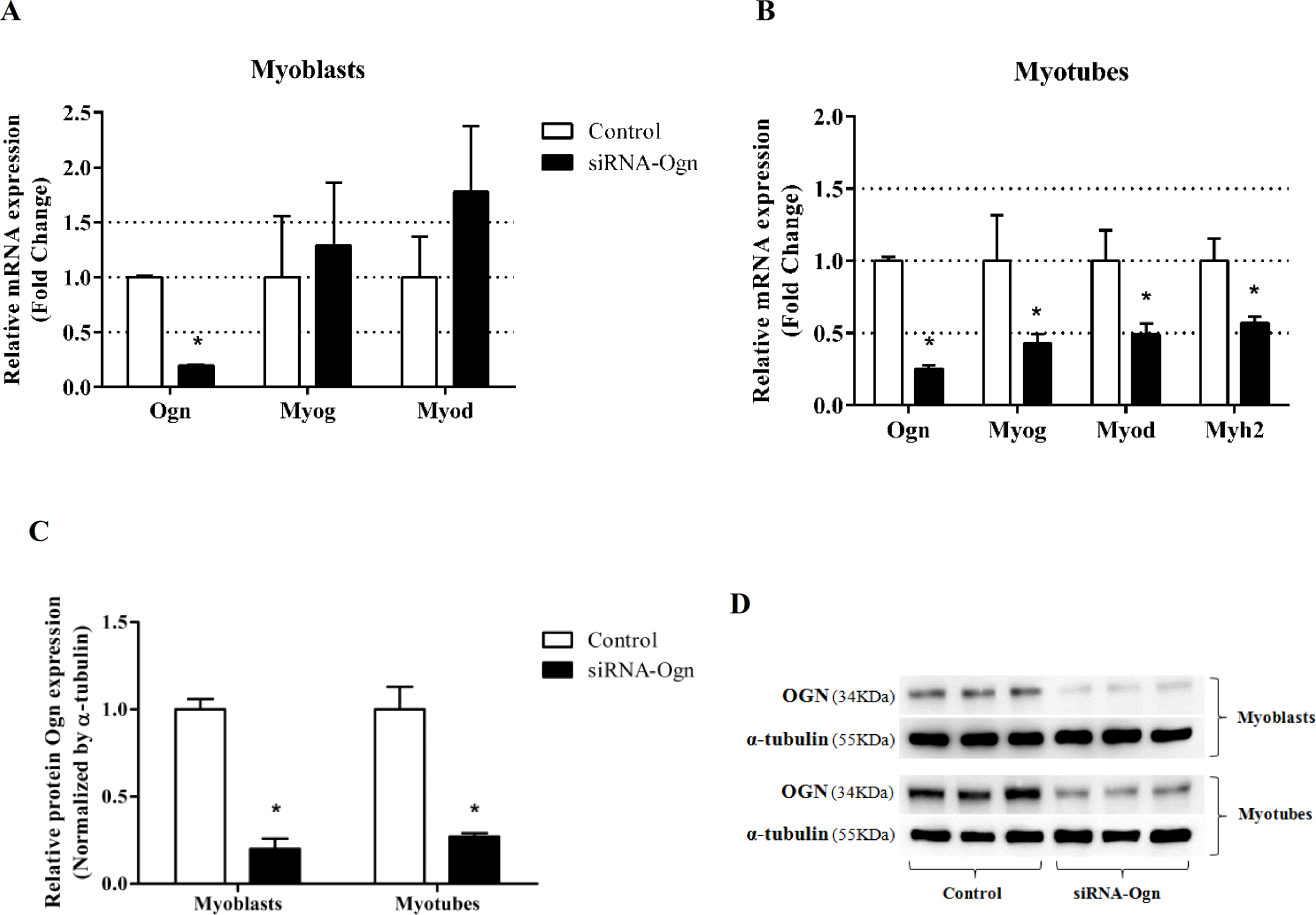
Ogn alters the expression levels of myogenesis molecular markers. (A) mRNA levels of Ogn, Myog, and Myod in myoblasts after Ogn knockdown. (B) mRNA levels of Ogn, Myog, Myod and Myh2 in myotubes after Ogn knockdown. (C) Protein levels of Ogn in C2C12 myoblasts (0 hours) and myotubes (120 hours, post differentiation). (D) Representatives blots. mRNA RT-qPCR data are presented as fold change (2^-ΔΔCt^) relative to Rpl13a. Western blot data was normalized by α-tubulin. Data represents the average of three independent experiments, and bars represent the standard deviation. Statistical significance was analyzed by the Student’s t-tests. *P < 0.05. Ogn: osteoglycin; Rpl13a: ribosomal protein L13A; Myog: myogenin; Myod: myogenic differentiation 1; Myh2: Myosin heavy chain II.

To test the functional relevance of Ogn in myogenesis, we knocked-down Ogn in C2C12 cells with a siRNA oligo. The Ogn knockdown was confirmed in both Ogn mRNA and protein levels in myoblast and myotubes (Fig 3A, 3B and 3C), although Ogn knockdown in myoblasts had no effect on Myod and Myog mRNA expression levels. In myotubes, siRNA-Ogn repressed myoblast differentiation as indicated by the reduced transcript levels of the myogenesis molecular markers Myog, Myod, and Myh2 (Fig 3B). These results indicate that Ogn alters the expression levels of myogenesis molecular markers that contribute to the differentiation of C2C12 cells.

### miR-155 and Ogn decreases C2C12 myoblasts proliferation

**Fig 4:**
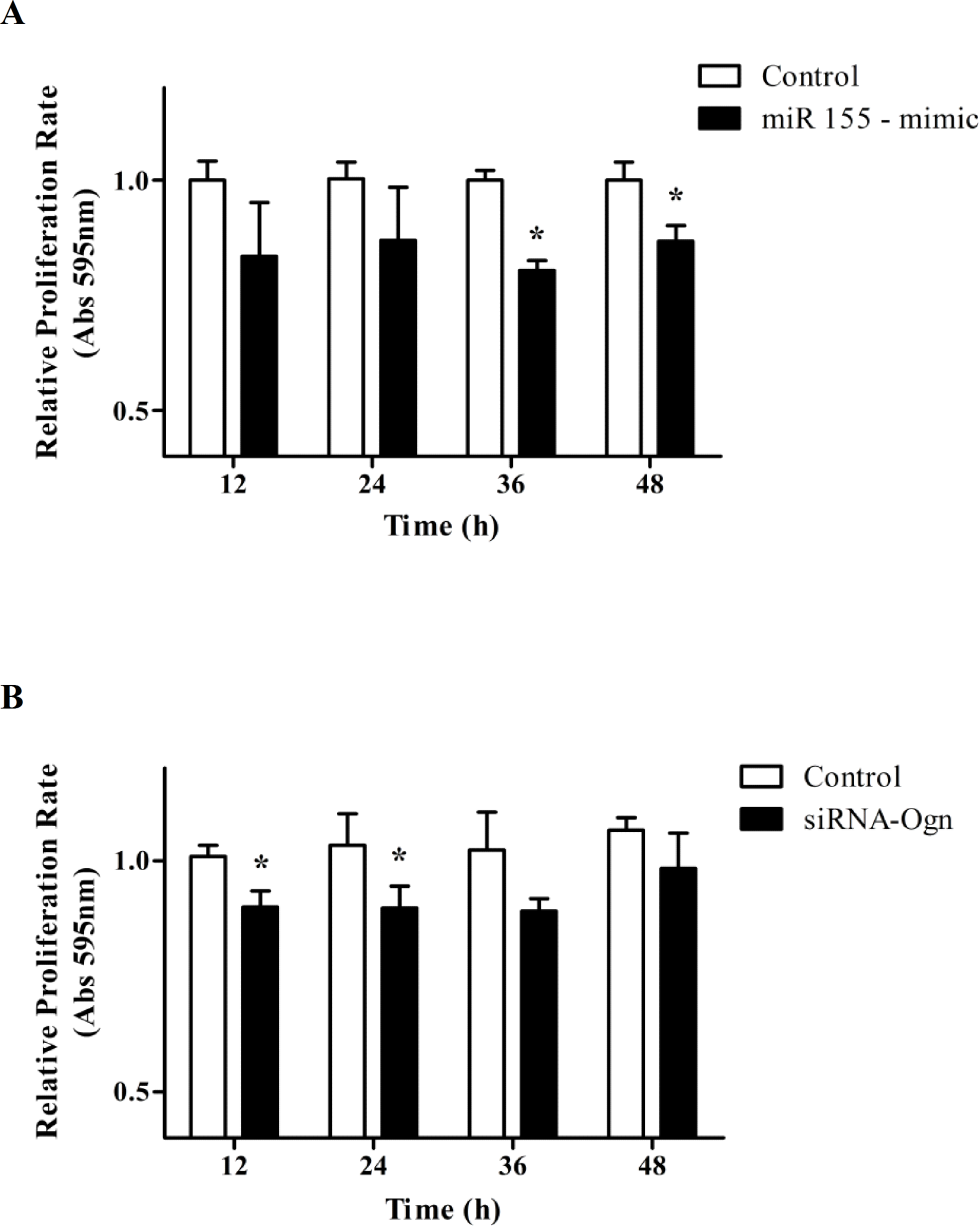
miR-155 and Ogn decreases C2C12 cell proliferation. Cellular proliferation rate analyzed by MTT assay after the transfection of C2C12 myoblasts with miR155-mimic (A) and siRNA-Ogn (B). Cellular proliferation rate was calculated considering the absorbance variation per hour between 12 hours, 24 hours, 36 hours, and 48 hours related to the initial absorbance (time 0 hours). The data represents the average of three independent experiments, and the bars represent the standard deviation. Statistical significance was analyzed by the Student’s t-tests. *P < 0.05.

To study the effects of miR-155 overexpression and Ogn-Knockdown on the C2C12 cells proliferation, miR-155 mimic or siRNA-Ogn were transfected into C2C12 cells. The C2C12 cell proliferation rate was measured using an MTT assay. The miR-155 overexpression in myoblast significantly decreased the proliferation rate at 36 and 48 hours (Fig 4A), while Ogn knocked-down in these cells induced a decreased proliferation rate at 12 and 24 hours. (Fig 4B). These results show that C2C12 cell proliferation rate is reduced by miR-155 overexpression and Ogn knockdown.

### miR-155 decreases C2C12 myoblasts wound closure rate

**Fig 5:**
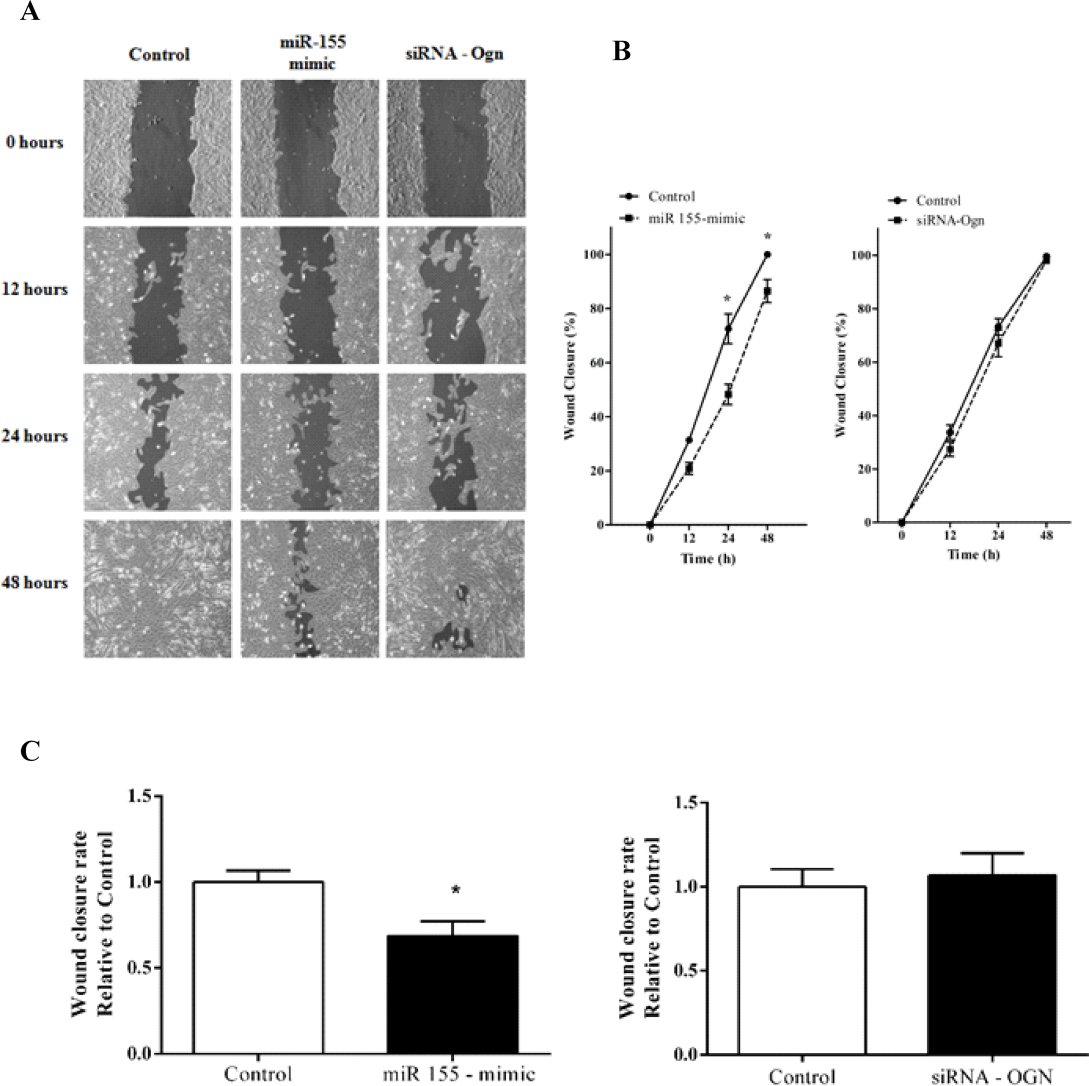
miR-155 decreases C2C12 cells wound closure. Cellular wound closure analyzed after the transfection of C2C12 myoblasts with miR155-mimic and siRNA-Ogn after 0 hours, 12 hours, 24 hours, and 48 hours post-wound (A and B). Wound closure was quantified by measuring the remaining unmigrated area and is expressed as a relative percentage (%).(C) Data was also expressed as wound closure rate per hour over the first 24 hours. The data represents the average of three independent experiments, and the bars represent the standard deviation. The statistical significance was analyzed using the Student’s t-tests. *P < 0.05.

After transferring miR-155 mimic and siRNA-Ogn, the distance between edges of the injured monolayer was measured at 0 hours, 12 hours, 24 hours and 48 hours after wounding (Fig 5A). C2C12 myoblasts exposed to miR-155 mimic showed a significant decrease in percentage of wound closure as demonstrated by the reduced at 24 hours and 48 hours with respect to controls (Fig 5B). This data was also confirmed by the wound closure rate per hour over the first 24 hours in the C2C12 myoblasts overexpression miR-155 (Fig 5C). The Ogn knockdown had no effect on C2C12 myoblasts wound closure (Fig 5B).

### miR-155 mimic and siRNA-Ogn inhibits C2C12 myotubes formation

**Fig 6:**
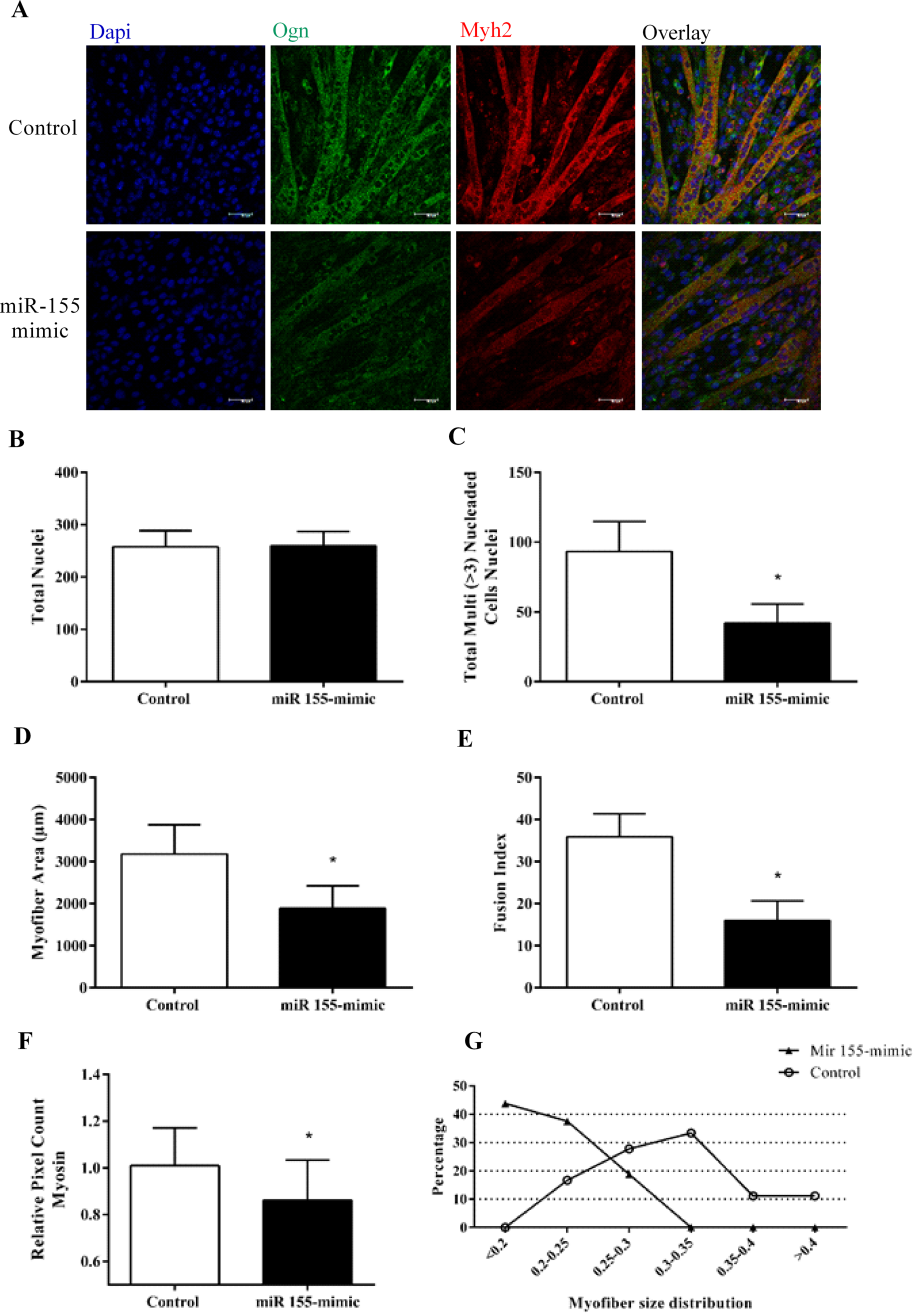
miR-155 inhibits C2C12 myotube formation. (A) Immunofluorescence of C2C12 myotubes transfected with miR 155-mimic stained with antibodies that recognize myosin heavy chain Myh2 (red) and osteoglycin Ogn (green). DAPI-stained nuclei. (B) Number of total nuclei. (C) Number of total myotube nuclei (three or more nuclei) (D) Quantitative analyses of myotube cell size in miR-155 overexpressed C2C12, by measuring the fiber area using ImageJ. (E) Myotube development is shown as fusion index (%) = (number of nuclei in myotubes)/(total number of nuclei in myoblasts and myotubes) x 100. (F) Myosin pixel count. (G) Distribution of myotube size. The data represents the mean ± standard deviation from at least three independent experiments. The statistical significance was analyzed using the Student’s t-test.

**Fig 7:**
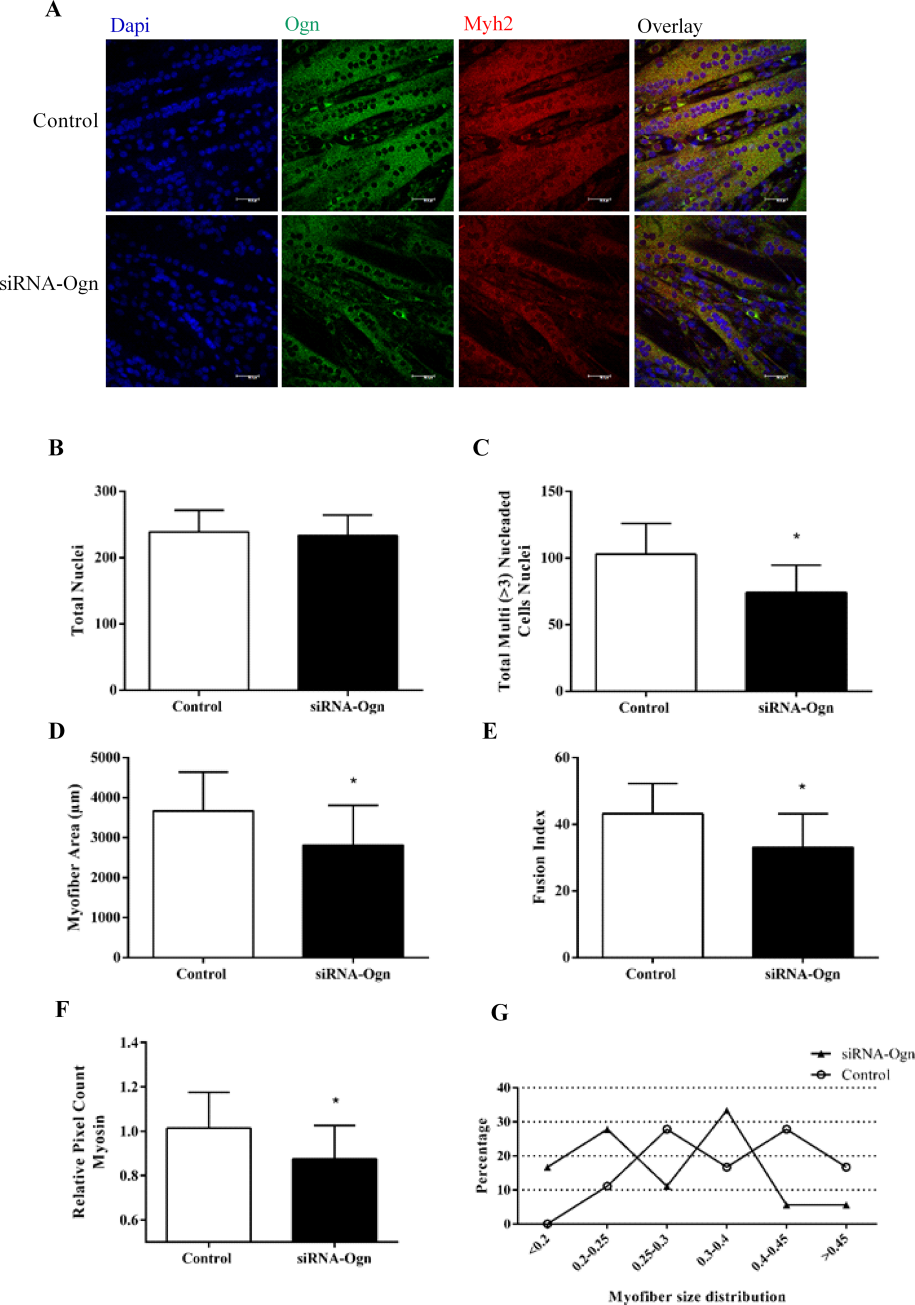
siRNA-Ogn inhibits C2C12 myotubes formation. (A) Immunofluorescence of C2C12 myotubes transfected with siRNA-Ogn stained with antibodies that recognize myosin heavy chain Myh2 (red) and osteoglycin Ogn (green). DAPI-stained nuclei. (B) Number of total nuclei. (C) Number of total myotube nuclei (three or more nuclei) (D) Quantitative analyses of myotube cell size in Ogn knockdown C2C12, by measuring the fiber area using ImageJ. (E) Myotube development is shown as fusion index (%) = (number of nuclei in myotubes) / (total number of nuclei in myoblasts and myotubes) x 100. (F) Myosin pixel count. (G) Distribution of myotube size. The data represents the mean ± standard deviation from at least three independent experiments. The statistical significance was analyzed using the Student’s t-test.

As a final step, the ability of miR-155 and Ogn to regulate myotube formation was investigated. C2C12 cells transfected with miR-155 mimic and with siRNA-Ogn had a lower ability to form myotubes compared to the controls (Figs 6 and 7). This analysis also revealed that both miR-155 and siRNA-Ogn transfections induced a significant reduction in number, area, fusion index, and Myh2 pixel count of multinucleated myotubes.

## Discussion

The main finding from our study was the identification and validation of a novel pathway in which miR-155 directly inhibits Ogn expression to regulate proliferation and differentiation of C2C12 cells during in vitro myogenesis. Firstly, we identified Ogn transcript as a putative miR-155 target on its 3‘UTR using five well-established miRNA-target prediction bioinformatics algorithms. Next, we evaluated the expression profile of Ogn and miR-155 in C2C12 cells during myogenesis (myoblasts and myotubes). Interestingly, Ogn protein levels inversely correlated with miR-155; decreased Ogn levels were concomitant with an increased miR-155 expression in myoblasts, and this pattern was inversely proportional in myotubes. This analysis strongly suggested that miR-155 represses the expression of Ogn by directly targeting its 3‘UTR region, which was further confirmed using a luciferase reporter gene assay. We also verified that miR-155 transfection in C2C12 cells induces a depletion in the Ogn mRNA and protein levels. To the best of our knowledge, there are no studies that have validated this miR-155/Ogn interaction, and this miR-155-induced Ogn depletion reduced the myoblast proliferation and differentiation. These effects were confirmed by knocking-down the Ogn expression which mimicked the effects of the miR-155 overexpression.

To examine whether miR-155 participates in the early processes of myogenesis, we transfected a mimetic miR-155 oligonucleotide into C2C12 myoblasts, which decreased myoblast proliferation after 36 and 48 hours of miR-155 transfection. Consistent with these results, we also found an increased myogenin gene expression in C2C12 myoblasts transfected with miR-155. Myogenin is a myogenic factor which increases its expression in the later stages of myogenesis [3,48], suggesting that miR-155 transfected myoblast cease proliferation prematurely to advance to the next steps of the myogenesis. This is different to Seok et al., 2009 [22], who showed that C2C12 myoblasts overexpressing miR-155 does not alter myogenin expression; however, these authors did not assess the proliferation rate of C2C12 myoblasts overexpressing miR-155. Moreover, genetic deletion of miR-155 also does not appear to directly regulate the proliferation or differentiation of miR-155-KO myoblasts, as supported by the expression of myogenic factors [20]. What could account for the disparities in the role of miR-155 in skeletal muscle cells proliferation? Firstly, these discrepancies may be associated with different gain- and loss-of-function experiments in myoblasts, which induce distinct phenotypes in these cells. Another possible explanation is the effect of the different experimental conditions under which miR-155 was transfected to myoblast, or how myogenin expression was assessed.

Interestingly, our results showing that miR-155 transfected myoblast reduces proliferation are supported further by an Ogn-siRNA induced Ogn gene silencing, which clearly reduced myoblast proliferation after 12–24 hours. This data is consistent with Hamajima, et al. 2003 [49], who showed that the increased expression of Ogn is associated with other effectors or extracellular matrix molecules that have important effects in proliferation and differentiation of MC3T3-E1 cells. This is different to Cui X et al. 2008 [50] who showed that Ogn did not influence the proliferative ability of mouse hepatoma Hca-F cells.

We also investigated whether myoblasts transfected with miR-155 have alterations in wound closure rate as an undirected measure of cellular migration and/or proliferation. These analyses revealed that miR-155 transfected myoblasts decrease wound closure after 24 hours and 48 hours compared to the respective control groups. Interestingly, miR-155 has been causally linked to cellular migration and proliferation in different cellular models. Previous in vitro analyses showed that miR-155 inhibited cell migration of human cardiomyocyte progenitor cells via targeting of MMP-16 [51] and, in pancreatic cancer cell, miR-155 plays an important role in the regulation of cell migration by modulating the STAT3 signaling pathway [52]. Importantly, the Ogn-siRNA induced gene silencing did not mimic the effects of the miR-155 overexpression in myoblast wound closure rate. Thus, it is important to consider the global impact of the miR-155-mediated regulation by targeting different mRNAs, which challenge the conceptualization of how cellular changes emerge from the effects of the miR-155 at the transcript level in skeletal muscle cells.

Lastly, to test whether miR-155/Ogn is involved in regulating the myoblast differentiation to myotubes, we evaluated the myogenesis molecular markers in myotubes transfected with miR-155 or silencing Ogn by siRNA. Our results showed a decrease in Myh2 transcript and protein expression levels after miR-155 mimic transfection, and the Ogn knockdown triggered a decrease in gene expression of Myog, Myod and Myh2, suggesting that Ogn is an important factor for skeletal muscle development. These results are coherent with those presented by Chan et al. 2011 [34] which found that Ogn knockdown inhibited myoblast differentiation, demonstrated by lower Muscle Creatine Kinase transcriptional activity. Our results expand these findings by showing an alteration in important myogenesis molecular markers such as Myod, Myog, and Myh2. These results are also supported by morphometric data demonstrating that miR-155 overexpression reduces myotube formation and size. Our results are similar to those obtained by Seok et al. 2011 [22] which demonstrated an inhibition of myoblast differentiation after miR-155 overexpression decrease the size and number of myotubes.

## Conclusion

In conclusion, we show that miR-155 directly targeted Ogn mRNA at the translational level to regulate proliferation and differentiation of C2C12 muscle cells during in vitro myogenesis.

## Supporting information

S1 Fig Geoprofile dataset. (PDF)

S2 Fig Experimental design and lipofectamine treatment. (PDF)

S3 Fig Oligonucleotides segment containing the miR-155 binding site on Ogn 3’UTR predicted by TargetScan algorithm. (PDF)

S4 Fig miR-155 alignment sequence in different species. (PDF)

S1 Table Genes and primer sequences used in RT-qPCR. (PDF)

S2 Table Reference genes used in RT-qPCR. (PDF)
