# Supplementary material for: Osteoglycin inhibition by microRNA miR-155 impairs myogenesis
Source: PLoS One. 2017 Nov 21;12(11):e0188464. doi: 10.1371/journal.pone.0188464 (PMC5697837; doi:10.1371/journal.pone.0188464)

Supporting Data

S1 Fig. Geoprofile Dataset

Increase in Ogn expression during the muscle regeneration after 12 hours, 1 day, 2 days, 4 days and 10 days.

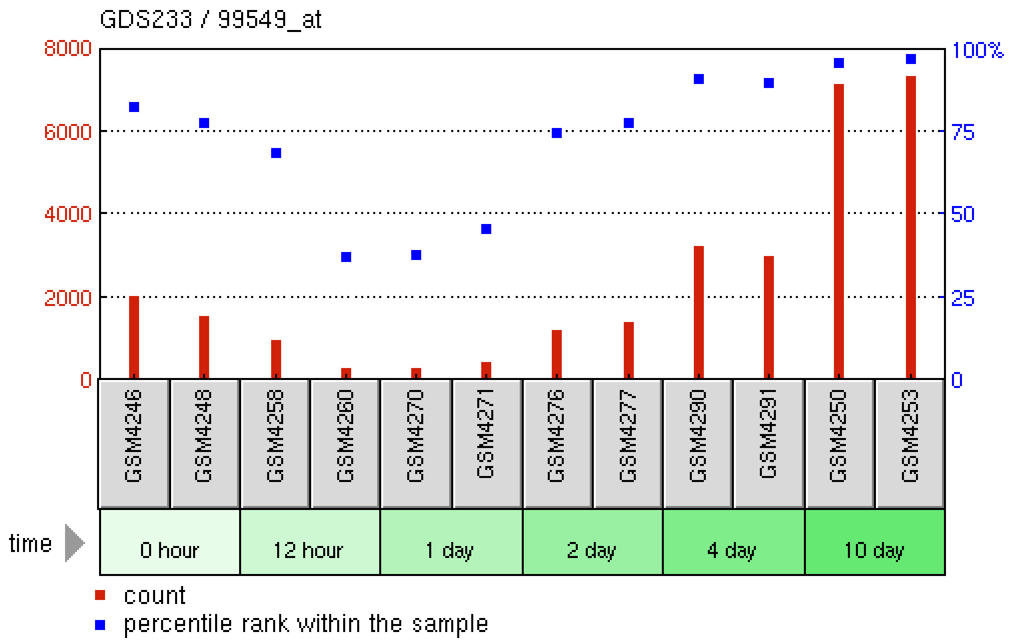

Supplement: S1 Fig — (PDF) [file pone.0188464.s001.pdf]
