# Supplementary figures and images for: Osteoglycin inhibition by microRNA miR-155 impairs myogenesis
Source: PLoS One. 2017 Nov 21;12(11):e0188464. doi: 10.1371/journal.pone.0188464 (PMC5697837; doi:10.1371/journal.pone.0188464)

# Supporting Data

**S2 Fig.**  
**Experimental design and lipofectamine treatment**

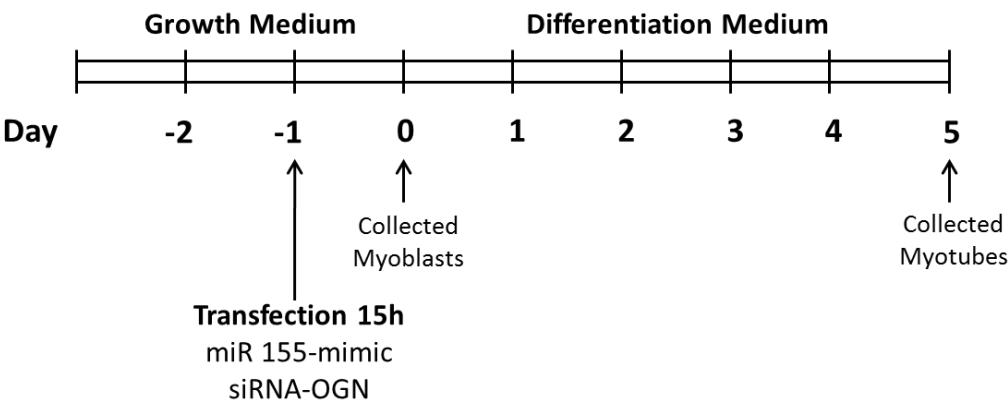

Supplement: S2 Fig — (PDF) [file pone.0188464.s002.pdf]
