# Supplementary material for: Osteoglycin inhibition by microRNA miR-155 impairs myogenesis
Source: PLoS One. 2017 Nov 21;12(11):e0188464. doi: 10.1371/journal.pone.0188464 (PMC5697837; doi:10.1371/journal.pone.0188464)
Supplement: S3 Fig — (PDF) [file pone.0188464.s003.pdf]

Supporting Data

S3 Fig.

Oligonucleotides segment containing the miR-155 binding site on Ogn 3'UTR predicted by TargetScan algorithm.

A

```
          DNA OGN 3' UTR 5'  ...ACTGTGAAACTAAACAGCATTAA...
   194-201nt OGN 3' UTR 5'  ...ACUGUGAAACUAAACAGCAUUAA...
                                               |||||||
              mmu-miR-155      UGGGGAUAGUGUUAAUCGUAAUU
```

B

OGN 3'UTR WT

```
1)OGN-3'UTR-Fw-wt   TCGAGTAGCGGCCGCTAGTACTGTGAAACTAAACAGCATTAAT
2)OGN-3'UTR-Rev-wt  CTAGATTAATGCTGTTTAGTTTCACAGTACTAGCGGCCGCTAC

 5'  TCGAG TA GCGGCCGC TAGT ACTGTGAAACTAAACAGCATTAA T      3'
         |  ||  ||||||||  ||||  |||||||||||||||||||||||
 3'      C  AT  CGCCGGCG  ATCA  TGACACTTTGATTTGTCGTAATT AGATC 5'
```

*Xho*I *Not*I miR-155 binding site *Xba*I

OGN 3'UTR Mut

```
1)OGN-3'UTR-Fw-mut   TCGAGTAGCGGCCGCTAGTACTGTGAAACTAAACAcgtaatAT
2)OGN-3'UTR-Rev-mut  CTAGATTgcattaGTTTAGTTTCACAGTACTAGCGGCCGCTAC

 5'  TCGAG TA GCGGCCGC TAGT ACTGTGAAACTAAACAcgtaatA T      3'
         |  ||  ||||||||  ||||  ||||||||||||||||******|
 3'      C  AT  CGCCGGCG  ATCA  TGACACTTTGATTTGTgcattaT AGATC 5'
```

*Xho*I *Not*I miR-155 binding site *Xba*I
