# Supplementary material for: Osteoglycin inhibition by microRNA miR-155 impairs myogenesis
Source: PLoS One. 2017 Nov 21;12(11):e0188464. doi: 10.1371/journal.pone.0188464 (PMC5697837; doi:10.1371/journal.pone.0188464)
Supplement: S4 Fig — (PDF) [file pone.0188464.s004.pdf]

Supporting Data

S4 Fig.

miR-155 alignment sequence in different species.

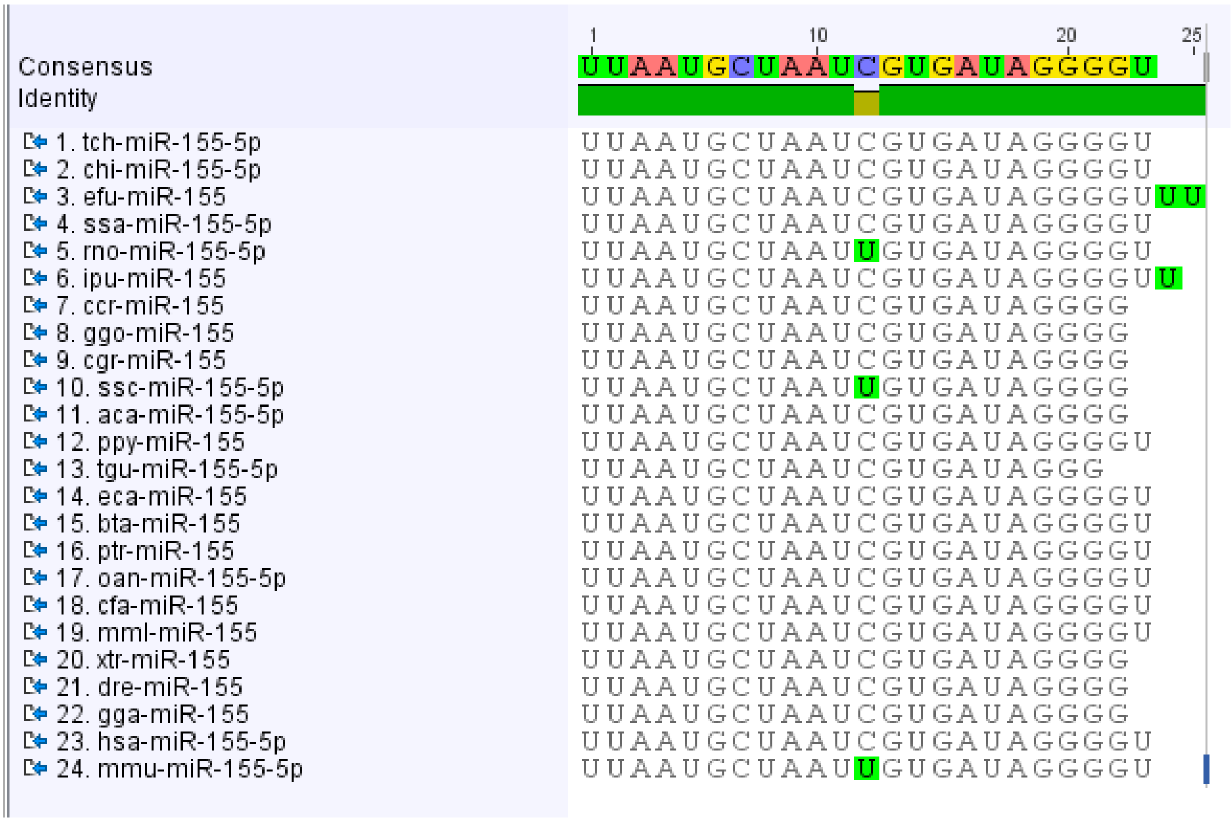

miRNA names are described according to the miRBase database which uses abbreviated 3 letter prefixes to designate the species. tch, *Tupaia chinensis*; chi, *Capra hircus*; efu, *Eptesicus fuscus*; ssa, *Salmo salar*; rno, *Rattus norvegicus*; ipu, *Ictalurus punctatus*; ccr, *Cyprinus carpio*; ggo, *Gorilla gorilla*; cgr, *Cricetulus griseus*; ssc, *Sus scrofa*; aca, *Anolis carolinensis*; ppy, *Pongo pygmaeus*; tgu, *Taeniopygia guttata*; eca, *Equus caballus*; bta, *Bos taurus*; ptr, *Pan troglodytes*; oan, *Ornithorhynchus anatinus*; cfa, *Canis familiaris*; mml, *Macaca mulatta*; xtr, *Xenopus tropicalis*; dre, *Danio rerio*; gga, *Gallus gallus*; hsa, *Homo sapiens*; mmu, *Mus musculus*.
