# Supplementary material for: Osteoglycin inhibition by microRNA miR-155 impairs myogenesis
Source: PLoS One. 2017 Nov 21;12(11):e0188464. doi: 10.1371/journal.pone.0188464 (PMC5697837; doi:10.1371/journal.pone.0188464)
Supplement: S1 Table — (PDF) [file pone.0188464.s005.pdf]

Supporting Data

S1 Table.

Genes and primer sequences used in RT-qPCR

Gene	Official Name	NCBI REF Seq	Sequence (5’-3’)
Myh2	*Mus musculus myosin, heavy polypeptide 2*	NM_001039545.2	CGAAGAGTAAGGCTGTCCCG<br>GCGCATGACCAAAGGTTTCA
Ogn	*Mus musculus osteoglycin*	NM_008760.4	AATGATGAAATGCCCACATGCC<br>TTTGGCAATGGTGGTACAGC
Myod1	*Mus musculus myogenic differentiation 1*	NM_010866.2	TCCTCATAGCACAGGGGTGA<br>GCAAGCTGTGGGGAAAAGTG
Myog	*Mus musculus myogenin*	NM_031189.2	CAGCCCAGCGAGGGAATTTA<br>AGAAGCTCCTGAGTTTGCCC
Rpl13a	*Mus musculus ribosomal protein L13A*	NM_009438.5	AACGGACTCCTGGTGTGAAC<br>TGGTCCCCACTTCCCTAGTT
Actb	*Mus musculus actin, beta*	NM_007393.5	CGTTGACATCCGTAAAGACC<br>TAGGAGCCAGAGCAGTAATC
Tbp	*Mus musculus TATA box binding protein*	NM_013684.3	GAGTTGTGCCAGAAGTTGGGC<br>CTTCACATCACAGCTCCCCA

NCBI REF Seq NCBI (http://www.ncbi.nlm.nih.gov/)
