# Supplementary material for: Osteoglycin inhibition by microRNA miR-155 impairs myogenesis
Source: PLoS One. 2017 Nov 21;12(11):e0188464. doi: 10.1371/journal.pone.0188464 (PMC5697837; doi:10.1371/journal.pone.0188464)
Supplement: S2 Table — (PDF) [file pone.0188464.s006.pdf]

Supporting Data

S2 Table. Reference genes used in RT-qPCR.

Transcript		Mim MB	CTm MB	Mim MT	CTm MT
U6	**Mean CT**	14.446	14.405	15.518	15.431
	**SD**	0.139	0.126	0.190	0.317
	**% variation**	0.965	0.874	1.221	2.055
Rpl13a*	**Mean CT**	18.197	18.178	18.989	19.044
	**SD**	0.160	0.295	0.112	0.044
	**% variation**	0.877	1.624	0.592	0.230

Mim MB: Myoblasts treated with mir-155 mimic; CTm MB: Control myoblasts; Mim MT: Myotubes treated with mir-155 mimic; CTm MT: Control myotubes.

* We performed an experiment, based on geNorm calculations, to identify the average expression stability (M-value) of three reference genes (Actb, Tbp, and Rpl13a) that revealed the following M-values for these genes: 0.5449, 0.4305, and 0.4236, respectively. Thus, this experiment reveal Rpl13a as the most stable reference gene.
